# miR-150: targeting MLL leukemia

**DOI:** 10.18632/oncotarget.736

**Published:** 2012-11-14

**Authors:** Xi Jiang, Jianjun Chen

**Affiliations:** Section of Hematology/Oncology, Department of Medicine, University of Chicago, Chicago, IL, USA; Section of Hematology/Oncology, Department of Medicine, University of Chicago, Chicago, IL, USA

It has been a tough challenge to hunt for effective therapeutic targets for acute leukemia bearing mixed lineage leukemia (*MLL*) fusion genes. The *MLL* gene which maps to human chromosome 11 band q23 (11q23) is frequently involved in chromosome translocations in around 10% of total leukemia, including 5-10% of patients with acute myeloid leukemia (AML) and 80% of infant acute leukemia. The critical feature of *MLL*-rearrangements is the generation of a chimeric transcript consisting of 5' *MLL* and 3' sequences of the partner gene; at present, more than 100 different loci which translocate to the *MLL* gene have been identified and more than 60 different partner genes have been cloned [1]. *MLL*-rearranged leukemia is usually associated with intermediate to poor prognosis and refractory to conventional therapies [1]. Therefore, it is urgent to develop new therapeutic methods which have high efficacy and low side-effects to treat *MLL*-rearranged leukemia.

These *MLL*-fusions are the “drivers” that determine the pathogenesis of the *MLL*-associated leukemias. Several important oncogenes (e.g. homeobox A (*HOXA*) genes, *MEIS1*, *FLT3*, *MYB* and *MYC*, etc.) serve as direct or indirect downstream targets of *MLL*-fusion proteins and are frequently up-regulated in *MLL*-associated leukemias. In addition to sharing a common driver and contributing to the pathogenesis of *MLL*-fusion-induced leukemia, these oncogenes have complex mutual regulatory influences on each other. For example, the *HOXA* cluster genes are the most well-defined direct targets of *MLL* fusions; *MYB* is an essential downstream target of the *HOXA*9/*MEIS1* signaling, and an autoregulatory feedback loop was reported recently in which *MYB* binds *MLL* through MENIN and regulates expression of *HOXA*9/*MEIS1* directly [2]; *MYC*, a potent oncogene which transcriptionally activates many tumor-promoting genes [3], is a downstream target gene of *MLL* fusions and *FLT3* [4]; *LIN28* is a direct target of *MYC* [5]. Taken together, a functional circuit, “driven” by *MLL*-fusions and involving all the above oncogenes (i.e. *HOXA*9, *MEIS1*, *FLT3*, *MYB* and *MYC*), exists in *MLL*-associated leukemia. However, how the *MLL*-pathway is regulated is unclear and this question merits deep exploration.

MicroRNAs (miRNAs), a class of small (22~25 bp) non-coding RNAs important in mediating post-transcriptional gene silencing, are involved in the pathogenesis of various types of cancers [1]. Jiang et al. [6] recently show that miR-150, a miRNA significantly down-regulated in most AML cases, is up-regulated by *MLL*-fusion/*MYC* at the primary transcription level but strikingly, is repressed by *MYC*/*LIN28* during the maturation process. miR-150 functions as a critical tumor suppressor in inhibiting *MLL*-fusion-induced cell transformation and leukemogenesis, through directly targeting *MYB*/*FLT3* and thereby subsequently interfering with the *HOXA*9/*MEIS1*/*FLT3*/*MYB*/*MYC*/*LIN28* signaling network. Collectively, this report describes a previously unappreciated regulatory circuit, namely *MLL*-fusion/*MYC*/*LIN28*⊣miR-150⊣ *FLT3*/*MYB*/*HOXA*9/*MEIS1* in *MLL*-associated leukemia, where miR-150 functions as a pivotal tumor-suppressing gate keeper [6].

During the past two decades, many efforts have been made to identify effective therapies targeting oncogenic components of the above circuit, e.g. inhibitors for *FLT3* and *MYC* [7, 8]. Given the essential role of miR-150 in inhibiting *MLL*-fusion-mediated cell transformation and leukemogenesis, and its remarkable repression of expression of all major downstream oncogenic targets of *MLL* fusions, restoring miR-150 expression/function holds great therapeutic potential in treating *MLL*-associated leukemia. Its side effect on normal hematopoiesis is likely weak, as overexpression of miR-150 in normal bone marrow progenitor cells results in only slight, if any, inhibitory effect on cell proliferation [6]. Thus, restoring the function of miR-150 by miRNA replacement (e.g. packaged in nano-particles, etc.) may provide an effective therapeutic strategy to treat *MLL*-associated leukemia, either alone or in combination with suitable chemotherapeutic drugs, or through synergy with future available therapeutic compounds that specifically target critical component(s) of the *MLL*-regulatory circuit such as *FLT3* or *MYC*.

Given the fact that miR-150 is significantly downregulated in almost all subtypes of AML and its forced expression exhibits a significant inhibitory effect on cell transformation mediated by various types of leukemic fusion genes, it would be important in the future to systematically investigate miR-150's pathologenic role and critical target genes/pathways in other subtypes of AML. It is also critical to determine whether the *MYC*/*LIN28*⊣miR-150⊣*FLT3*/*MYB*/*HOXA*9/*MEIS1* regulatory circuit also exists and is functionally important as a whole or at least in part in other subtypes of leukemia, or even other types of cancers (e.g., lymphomas). Such studies may lead to the ultimate development of highly effective targeted therapy approaches to treat not only *MLL*-associated leukemia, but also other subtypes of leukemia or even other types of cancers that also utilize at least part of the aforementioned signaling circuit.

The authors thank Dr. Janet D. Rowley for her critical comments.
